# Chinese Eye Exercises and Myopia Development in School Age Children: A Nested Case-control Study

**DOI:** 10.1038/srep28531

**Published:** 2016-06-22

**Authors:** Meng-Tian Kang, Shi-Ming Li, Xiaoxia Peng, Lei Li, Anran Ran, Bo Meng, Yunyun Sun, Luo-Ru Liu, He Li, Michel Millodot, Ningli Wang

**Affiliations:** 1 Beijing Tongren Eye Center, Beijing Tongren Hospital, Beijing Ophthalmology Visual Science Key Lab, Beijing Institute of Ophthalmology, Capital Medical University, Beijing, China; 2 Department of Epidemiology and Biostatistics, School of Public Health and Family Medicine, Capital Medical University, Beijing, China; 3 Anyang Eye Hospital, Henan Province, China; 4 School of Optometry and Vision Sciences, Cardiff University, Cardiff, United Kingdom

## Abstract

Chinese eye exercises have been implemented in China as an intervention for controlling children’s myopia for over 50 years. This nested case-control study investigated Chinese eye exercises and their association with myopia development in junior middle school children. Outcome measures were the onset and progression of myopia over a two-year period. Cases were defined as 1. Myopia onset (cycloplegic spherical equivalent ≤ −0.5 diopter in non-myopic children). 2. Myopia progression (myopia shift of ≥1.0 diopter in those who were myopic at baseline). Two independent investigators assessed the quality of Chinese eye exercises performance at the end of the follow-up period. Of 260 children at baseline (mean age was 12.7 ± 0.5 years), 201 were eligible for this study. There was no association between eye exercises and the risk of myopia-onset (OR = 0.73, 95%CI: 0.24–2.21), nor myopia progression (OR = 0.79, 95%CI: 0.41–1.53). The group who performed high quality exercises had a slightly lower myopia progression of 0.15 D than the children who did not perform the exercise over a period of 2 years. However, the limited sample size, low dosage and performance quality of Chinese eye exercises in children did not result in statistical significance and require further studies.

Myopia is a major health issue in East Asia, because of its increasingly high prevalence during the past few decades, and its sight-threatening pathologies associated with high myopia^1^. The current status of myopia in China has also raised a great deal of attention due to the young age of myopia onset and an increasing prevalence of high myopia in children^2^. As we know, young children have a progressive nature of myopia development and are sensitive to environmental factors^3^,^4^,^5^. Although myopia tends to stabilize at approximately 16 years of age^6^, children with a younger age of myopia onset are prone to becoming highly myopic at an early stage of life and may suffer from complications. Therefore, it is important to find simple and easy-to-use interventions, not limited to current methods of eye drops^7^ or lenses^8^,^9^, to prevent myopia onset or to slow myopia progression in young children.

Chinese eye exercises have been implemented in China as an intervention for protecting vision and preventing myopia of children since 1963^10^,^11^. Children in primary and junior middle schools have been required to perform eye exercises once or twice a day. When the music starts during class break, all students sit in the classroom and perform 05-min exercises along with the rhythm. The mechanism of eye exercises is believed to be based on the theory of Traditional Chinese Medicine (TCM). By precise massage on acupuncture acupoints, Qi can be motivated in meridians and collaterals, thereby relieving eye strain and recovering eye function^12^,^13^. From the perspective of modern medicine, massaging on the acupoints around the eyes will accelerate blood circulation and therefore will improve metabolism, relax eye muscles and eliminate eye fatigue^14^.

In spite of the long history of their popularization, the elusive effect of Chinese eye exercises on myopia has attracted increasing attention during the last decade^15^. A retrospective study reported that Chinese eye exercises have a modest effect on relieving near vision symptoms but no remarkable effect on reducing myopia^16^. Some cross-sectional studies have found different associations of myopia prevalence with eye exercises^17^,^18^. It has also been reported that having a “serious attitude” toward performing these exercises improved visual acuity^19^. However, due to the lack of suitable evidence and lack of adjustments to the data (e.g. time spent in outdoor and near work, the number of parents with myopia, the age of myopia onset), it is still uncertain whether Chinese eye exercises are effective in controlling childhood myopia.

A randomized controlled trial (RCT) would be an ideal way to investigate the effect of Chinese eye exercises. But since eye exercises have become a daily ritual for half a century in China, it has not been possible to obtain the parents’ permission to perform a RCT by allocating some children to stop doing Chinese eye exercises for years, because it is generally believed that they are useful. Also, it would be difficult to get approval for a control group from an ethics committee in China. The best evidence is likely to come from a well-designed observational study, which adequately controls for potential confounding variables, to investigate the association between Chinese eye exercises and myopia.

It is also important to estimate the effect of the quality of performance on myopia. To assess a child’s use of Chinese eye exercises, a number of factors should be taken into account. First, not every child performs eye exercises during the allocated time set up by school. According to a previous survey^20^, many children thought it was boring to do eye exercises and preferred to use the time to study. In addition, many children were performing the eye exercises in a wrong way, which resulted in substandard eye exercises. A questionnaire survey^21^ of 3120 Guangzhou students in primary and junior middle schools found that 97% of school age children did not know the correct pressure to do eye exercises. 91% of children did not know the basic massage manipulation. In addition, 96% of children could not find the correct location of acupoints.

In a previous randomized control trial, we have found that Chinese eye exercises performed daily can reduce the accommodative lag of school age children in the short-term^22^. In the present nested case-control study for school age children during a follow-up period of 2 years, we aimed to address (1) whether Chinese eye exercises are associated with myopia progression or myopia onset; (2) whether the quality of eye exercises performance has an effect on myopia progression and axial elongation.

## Methods

### Sources of Data and Ethics Statement

The Anyang Childhood Eye Study (ACES)^2^,^23^ was a school-based cohort study conducted in Anyang city in central China. The ACES was designed to longitudinally observe the onset and development of myopia in school age children. The ocular examinations included visual acuity, testing for amblyopia and strabismus, ocular biometry, optical coherence tomography, retinal photography, cycloplegic autorefraction, etc.^24^,^25^. All investigations were performed in accordance with ACES guidelines and regulations. The ACES was approved by the Human Research Ethics Committee of the Tongren Hospital, Capital Medical University Beijing. The nature of the study was explained to parents and children and informed written consent was obtained from at least one parent.

### Study Design

The present study was a nested case-control study by selecting grade 7 children from the ACES and following them for 2 years (September 1, 2011 through December 31, 2013). From one randomly selected school in the ACES, 260 school age children were followed up during the study period. Inclusion criteria were: Participants who did not use any form of myopia control such as contact lenses, orthokeratology, acupuncture, massage, eye drops or ear needles; Best-corrected visual acuity ≥6/6 ; cycloplegic spherical equivalent refraction (SER) ranging from +0.50D to −6.00D ; astigmatism less than −1.50D ; anisometropia less than 1.0D; no history of ocular or systemic abnormalities which might affect visual function or refractive development. Children’s practice of Chinese eye exercises were assessed by questionnaire and the quality of performance was scored.

### Definition of Case and Control

Myopia was defined as cycloplegic SER ≤ −0.5D. Of the children who were myopic at baseline, those having myopic progression ≥1.0D during the 2 year follow-up period were defined as cases of myopia progression, whereas those who did not progress were defined as controls. Of the children who were not myopic at baseline, those having myopia onset (cycloplegic SER ≤ −0.5D) during the follow-up period were defined as cases, whereas those whose myopia did not appear during the study period were defined as controls.

### Eye Examinations and Questionnaires

Axial length and cycloplegic refraction were examined annually for 3 years. LenStar LS900 (Haag-Streit Koeniz, Switzerland) was used to measure axial length, anterior chamber depth, corneal curvature and lens thickness. Five repeated measurements were taken and averaged. An autorefractor (HUVITZ, HRK-7000A, South Korea) was used to measure cycloplegic refraction. Each child was first administered one drop of topical anesthetic agent (Alcaine, Alcon) to alleviate discomfort, followed by 2 drops of 1% cyclopentolate (Alcon) and 1 drop of Mydrin P (Santen, Japan) at 5-minute intervals. 30 minutes after the last drop, a third drop of cyclopentolate was administrated if the pupillary light reflex was still present or the pupil size was less than 6.0 mm. Five repeated measurements of spherocylindrical autorefraction were taken and averaged.

Both cases and controls were asked to complete a questionnaire to collect possible myopia related factors including number of parents with myopia, frequency of doing eye exercises, time spent in near work, outdoor activities, etc. The design of the questionnaires used in the ACES has been described elsewhere^2^.

### Exposure to Chinese Eye Exercises

To assess the quality of eye exercises, two independent investigators used the standard Chinese Eye Exercises assessment form (Supplemental File 1) to assess and score the quality of performance of each participant. The assessment of eye exercises was collected by 2013 (the second year of follow-up) and represented the past 2 years’ conditions. The assessment consisted of 40 items including massage location, force, scope, and frequency of massage, with one score for each item. On the basis of a total score of 40, scores greater than 30/40 and differences between two judges less than 5 were defined as qualified. The frequency of doing exercises per week was assessed by questionnaire. According to school rules, students should do eye exercises at least once a day (which means 5 times per week).

Based on the score of the performance and the frequency of doing exercises, we evaluated a child’s practice of eye exercises qualitatively and quantitatively. The use of Chinese eye exercises was stratified into three groups: 1.High quality group: children who had high score eye exercises (score ≥30) AND had eye exercises at least once a day. 2. Low quality group: children who had low score eye exercises (score <30) OR did not have exercises once a day. 3. No exercises: children never perform eye exercises.

Independent investigators scored eye exercises and tested SER and axial length. Outcome investigators and participants were all blinded to the design of this study.

### Statistical Analyses

Descriptive statistics were used to examine the covariate distribution between case and control. T-test and chi-square test were used to compare the covariate distribution of case and control groups. Unconditional logistic regression was used to estimate the odds ratios (ORs) and the corresponding 95% confidence intervals for the association between Chinese eye exercises and myopia progression/myopia onset. The following four statistical models were constructed: Crude model assessed the association between the quality of Chinese eye exercises and myopia progression without adjustment for confounding variables. Model 1 was adjusted for age and gender. Model 2 was further adjusted for number of myopic parents (Neither, one, both), time spent in near work and outdoor activities. Model 3 was further adjusted for baseline SER and axial length because there was a significant difference between myopia-onset in cases and controls. The age of myopia-onset was adjusted in the analysis of myopia progression in cases, because it had a significant relationships to myopia progression^26^. For Chinese eye exercises, stratified analysis was performed by groups (High quality, low quality and no exercise group) as described above. SPSS 20.0 software for Windows was used for all statistical analyses. All P-values were 2-sided and considered statistically significant when less than 0.05.

## Results

### Characteristics of Cases and Controls

**Figure 1:**
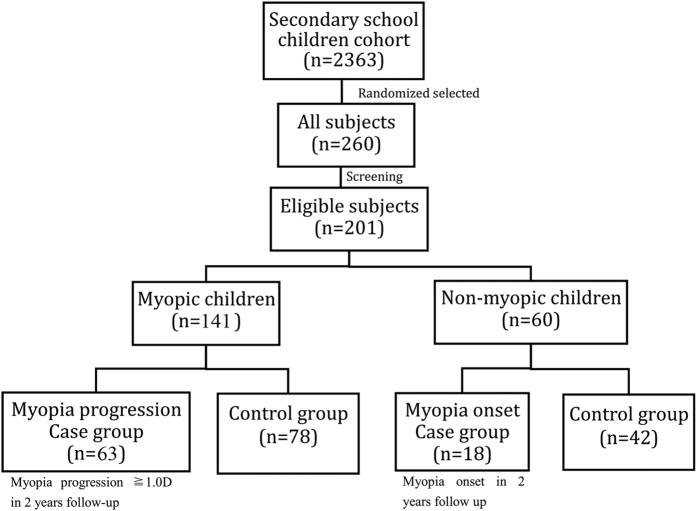
Flow chart of subjects in the nested case-control study.

During the study period, 260 children were followed up for 2 years and 201 were included for analysis (59 children were excluded because they did not fulfill the inclusion criteria described above). Of these 103 were males and 98 were females with mean age of 12.7 ± 0.5 years at baseline. Of 141 myopic children at baseline, 63 (44.7%) children who had myopia progression of more than 1.0D in the 2 years’ follow-up period were classified as myopia progression cases, and 78 (54.9%) children as myopia progression controls. Of 60 non-myopic children at baseline, 18 (30.0%) who had myopia onset were classified as myopia-onset cases, and 42 (70%) children as myopia-onset controls (Fig. 1).

At baseline, myopia-onset cases had longer axial length (24.0 ± 0.6 mm) and more myopic SER (0.08 ± 0.49D) compared to controls (23.1 ± 0.7 mm and 0.84 ± 0.88D, P < 0.01). During the follow up, myopia onset cases had more time spent in near work (4.31 ± 2.47 h/d) compared to controls (2.83 ± 1.13 h/d) (t = −3.191, P < 0.01). There was no statistical difference in age, number of parents with myopia, gender and time spent in outdoor activities between myopia onset cases and controls. There was no statistical difference in age, myopia onset age, number of parents with myopia, gender, time spent in nearworks and outdoor activities, axial length, SER between myopia progression cases and controls (Table 1).

### Association between Chinese Eye Exercises and the Risk of Myopia Development

We stratified the participants according to the frequency and the score of performance (Table 2). Overall, more than half of the children (51.2%) had never done eye exercises during the follow up. 33.3% of children achieved low quality eye exercises. Only a minority of children (15.4%) achieved high score eye exercises everyday (who were in high quality group). The percentage of children doing high quality eye exercises in the myopia onset case group was less than in the control group (5.6% vs. 26.2%), and in the myopia progression case group it was also less than in the control group (11.1% vs. 15.4%). However, the number of cases of high quality exercises performer was too small (n = 1 in myopia onset case and n = 7 in myopia progression cases) and could not be analyzed further in unconditional logistic regression. Thus, we estimated the odds ratios and the corresponding 95% confidence intervals for the associations between Chinese eye exercises and myopia progression/myopia onset.

The association of eye exercises with the risk of myopia onset was not statistically significant (OR = 0.73, 95%CI: 0.24–2.21). This association attenuated after adjustment for age and gender (OR = 0.81, 95%CI: 0.26–2.59), after further adjustment for time spent in near work, time spent in outdoor activities, number of parents with myopia (OR = 0.86, 95%CI: 0.51–1.44), and further adjustment for baseline SER and axial length (OR = 1.35, 95%CI: 0.52–2.56). There was also no apparent association between the practice of eye exercises and the risk of myopia progression compared to those who did not exercise (OR = 0.79, 95%CI: 0.41–1.53) (Table 3).

### Mean Change in Axial Length and Spherical Equivalent Refraction with Different Qualities of Eye Exercises Performance

**Figure 2:**
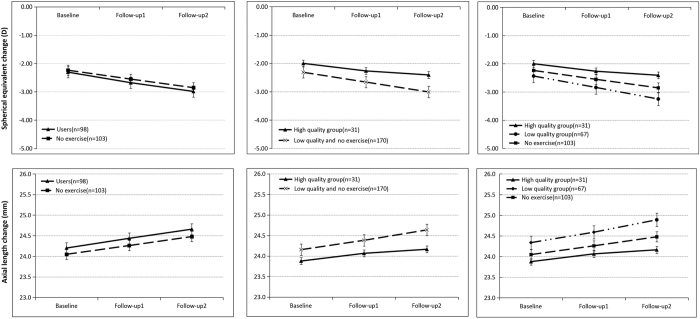
Annual change in axial length and spherical equivalent refraction with different qualities of eye exercises performance. Error bars mean standard error.

We compared the mean change of axial length and SER in children with high quality eye exercises, low quality eye exercises and without exercises. Figure 2 showed the annual change in axial length and SER with different qualities of eye exercises performance. All groups showed a trend of increasing axial length and more myopic SER during 2-year follow-up. At baseline, the mean axial length and SER had no statistically significant difference among high quality, low quality eye exercises and no exercise group. The mean change of SER and axial length also showed no statistically significant difference among the three groups (−0.47D vs. −0.76D vs. −0.62D) (0.4 mm vs. 0.5 mm vs. 0.4 mm) (P > 0.05) (Table 4). We got the same results after adjusting for age, gender, time spent in near work and outdoor activities, myopia onset age. When comparing the no exercise group with the high quality group the differences were 0.15D and 0.0 mm and they were −0.15D and 0.1 mm when compared to the low quality group.

## Discussion

It is of notable public health implications in China to investigate whether Chinese eye exercises have an effect on controlling myopia in children. Our previous study^22^ has demonstrated that Chinese eye exercises have a statistically significant effect on reducing the accommodative lag of children in the short-term, the children having been examined after performing the exercises only once. In the present study, we found that the children with high quality performance of the exercises had slightly less myopia progression rate than the children with low quality, although there was an insignificant association between Chinese eye exercises and the risk of myopia onset and myopia progression.

In subgroup analysis, the children in the high quality group had a smaller myopia progression of 0.15 D than the children who did not perform the exercise over a period of 2 years (Table 4). This difference seems to be clinically insignificant but was still comparable to the effect of outdoor activities. In a recent randomized controlled trial, He *et al*.^27^ found that children with an additional increase of 40 minutes of time outdoors a day had a slower myopia progression of 0.17 D than that of the controls during 3 year period.

In schools, most Chinese children were required to perform Chinese eye exercises only once or twice a day, once in the morning and once in the afternoon, with 5 minutes for each time. Therefore, the total time of performing Chinese eye exercises was at most 10 minutes a day and in some places the children only performed the exercise once a day. Overall, the dosage of Chinese eye exercises for children was very small regardless of whether it has a real effect on controlling myopia or not.

More importantly, most children could not perform the Chinese eye exercises with standard manipulation. Previous studies showed that about 90% of Chinese children did not perform the exercises correctly, most of them could not find the exact periocular acupoints and did not have accurate pressure and manipulation skills for the exercises although they did them every day^28^,^29^. In this study, only about 15% of total children and about a third of those who performed them were found to achieve high quality eye exercises. This greatly limits the possibility of detecting the real effect of Chinese eye exercises on controlling myopia progression although they are required to be performed by children every day.

The relatively small sample size in this study is another factor that might affect the power to detect the effect of Chinese eye exercises. We have included only 201 children which is very few compared with 1903 children in the large scale randomized controlled trial on outdoor activities. Chinese eye exercises are a form of massage on ocular acupoints which is even weaker than the acupuncture on acupoints. Therefore, an equal or even larger number of children than that for outdoor activities might be needed for Chinese eye exercises to detect their effect.

Still, to our knowledge, this is the first study that attempts to detect the effect of Chinese eye exercises on controlling myopia in children in the long-term. We hope that this study will give us some evidence on the effect of Chinese eye exercises and indicate what we should do in future. We used a design of nested case-control study, which is not an ideal design compared to randomized controlled trial but is the best solution that we can do in the current conditions. In China, it would be impossible to allocate some children to stop performing Chinese eye exercises for years as it would not be permitted by their parents, teachers or an ethics committee. We assume that an RCT would be easier to be conducted in other countries or areas, in which the schools did not require eye exercises as a routine regulation.

A cohort of primary school students will be investigated in the future, which should give a better chance of demonstrating the impact of the exercise on slowing progression. We plan to expand sample size in a larger study with the same design in primary school students in the next year. It is necessary to evaluate the status in primary school students, who are in the early stage of myopia progression, and use eye exercises in the same way as for junior high school students.

It should be noted that the results of our study can only be generalized to children of junior middle school. We could not extend the conclusion to younger children since they have different characteristics in eye growth. We chose junior middle school students because they have better expressing ability than primary school students. Children in a same grade will ensure homogeneity of age, because age is highly related to the rate of myopia progression. In addition, the children were assessed for their performance of Chinese eye exercises at the end of the follow-up period. The recall bias might cause decreased accuracy regarding the frequency of doing eye exercises.

In summary, children with high quality eye exercises seem to have a slightly lower myopia progression rate, but with limited sample size to detect a statistical significance. Further study will be conducted in a larger sample size and/or a randomized controlled trial to see whether Chinese eye exercises are effective in primary school children. Since neglecting hand hygiene might cause conjunctivitis^21^, instruction about standard procedures should also be generalized along with the school rules for performing eye exercises. Moreover, emphasis on changing indoor lifestyle^30^,^31^ and encouraging increasing outdoor activities^5^,^32^,^33^ should also be considered in China.

## Additional Information

**How to cite this article**: Kang, M.-T. *et al*. Chinese Eye Exercises and Myopia Development in School Age Children: A Nested Case-control Study. *Sci. Rep.* **6**, 28531; doi: 10.1038/srep28531 (2016).

## Supplementary Material

Supplementary Information

## Figures and Tables

**Table 1:** Baseline data of children who were not myopic (myopia onset) and myopic(myopia progression).

	All ((n = 201)	Myopia onset (n = 60)		Myopia progression (n = 141)	
		Cases* (n = 18)	Controls (n = 42)	Cases** (n = 63)	Controls (n = 78)
Age (y)	12.7 ± 0.5	12.7 ± 0.5	12.7 ± 0.7	12.7 ± 0.4	12.7 ± 0.4
Myopia onset age (y)	11.2 ± 1.8	13.2 ± 0.8	–	10.9 ± 1.7	11.0 ± 1.8
Gender (%)					
Male	103 (51.2)	8 (44.4)	27 (64.3)	37 (58.7)	31 (39.7)
Female	98 (48.8)	10 (55.6)	15 (35.7)	26 (41.2)	47 (60.3)
Number of parents with myopia (%)					
Neither	148 (73.6)	13 (72.2)	37 (88.1)	42 (66.7)	56 (71.8)
One	43 (21.4)	5 (27.8)	5 (11.9)	16 (25.4)	17 (21.8)
Both	10 (5.0)	0 (0.0)	0 (0.0)	5 (7.9)	5 (6.4)
Time near work (h/d)	3.41 ± 1.59	4.31 ± 2.47	2.83 ± 1.13	3.29 ± 1.40	3.58 ± 1.61
Time outdoor (h/d)	1.68 ± 1.99	1.50 ± 1.14	1.53 ± 1.00	1.83 ± 3.25	1.68 ± 1.11
Axial length (mm)	24.1 ± 1.0	24.0 ± 0.6	23.1 ± 0.7	24.5 ± 0.9	24.3 ± 0.9
Non-cycloplegic SER (D)	−2.27 ± 1.68	−1.10 ± 0.93	−0.55 ± 1.02	−3.04 ± 1.61	−2.79 ± 1.35
Cycloplegic SER (D)	−1.57 ± 1.87	0.08 ± 0.49	0.84 ± 0.88	−2.64 ± 1.53	−2.23 ± 1.35

SER = spherical equivalent refraction.

^*^Cases were non-myopic children who have myopia onset during the follow-up. Controls were non-myopic children who did not have myopia onset during the follow-up.

^**^Cases were myopic children who have myopic progression ≥1.0D during the follow-up. Controls were myopic children who have myopic progression <1.0D during the follow-up.

**Table 2:** Eye exercises use among cases and controls.

	All (n = 201)	Myopia onset (n = 60)		Myopia progression (n = 141)	
		Cases* (n = 18)	Controls (n = 42)	Cases** (n = 63)	Controls (n = 78)
Frequency					
≥5/week	44 (21.9)	2 (11.1)	12 (28.6)	14 (22.2)	16 (20.5)
1–4/week	54 (26.9)	8 (44.4)	8 (19.0)	18 (28.6)	20 (25.6)
No exercise	103 (51.2)	8 (44.4)	22 (52.4)	31 (49.2)	42 (53.8)
Performance score					
≥30	79 (39.3)	9 (50.0)	17 (40.5)	24 (38.1)	29 (37.2)
1–29	19 (9.5)	1 (5.6)	3 (7.1)	8 (12.7)	7 (9.0)
0	103 (51.2)	8 (44.4)	22 (52.4)	31 (49.2)	42 (53.8)
Group					
High quality	31 (15.4)	1 (5.6)	11 (26.2)	7 (11.1)	12 (15.4)
Low quality	67 (33.3)	9 (50.0)	9 (21.4)	25 (39.7)	24 (30.8)
No exercise	103 (51.2)	8 (44.4)	22 (52.4)	31 (49.2)	42 (53.8)

^*^Cases were non-myopic children who have myopia-onset during the follow-up. Controls were children who did not have myopia-onset during the follow-up.

^**^Cases were myopic children who have myopic progression ≥1.0D during the follow-up. Controls were myopic children who have myopic progression <1.0D during the follow-up.

**Table 3:** Odd ratios (ORs) and 95% confidence intervals for the association between eye exercises and the risk of myopia-onset/myopia progression.

Determinants	OR (95%CI)			
	Crude Model	Model 1	Model 2*	Model 3
Myopia-onset				
Eye exercises users	0.73 (0.24–2.21)	0.81 (0.26–2.59)	0.86 (0.51–1.44)	1.35 (0.52–2.56)
No exercise	referent	referent	referent	referent
Myopia progression				
Eye exercises users	0.79 (0.41–1.53)	0.88 (0.44–1.75)	0.63 (0.28–1.41)	0.64 (0.27–1.47)
No exercise	referent	referent	referent	referent

Crude model: Without adjustment.

Model 1: Adjusted for age, gender.

Model 2: Further adjusted for time spent in near work, time spent in outdoor activities, parental myopia.

Model 3: Further adjusted for baseline SER and axial length.

*Myopia-onset age was adjusted in myopia progression cases and controls.

**Table 4:** Characteristics distribution among all children with different qualities of eye exercises performance.

	Users of eye exercises(n = 98)		No exercise(n = 103)	P
	High quality(n = 31)	Low quality(n = 67)		
Age (y)	12.7 ± 0.5	12.7 ± 0.6	12.6 ± 0.5	0.800
Gender (%)				
Male	17 (54.8)	36 (53.7)	50 (48.5)	
Female	14 (45.2)	31 (46.3)	53 (51.5)	0.731
Time spent in near work (h/d)	3.71 ± 2.08	3.42 ± 1.43	3.31 ± 1.52	0.463
Time spent in outdoor activities (h/d)	1.53 ± 0.87	2.13 ± 3.09	1.43 ± 1.09	0.073
Myopia-onset age (y)	11.1 ± 1.4	11.4 ± 1.8	11.1 ± 1.9	0.758
Baseline axial length (mm)	23.9 ± 0.9	24.3 ± 1.0	24.0 ± 1.0	0.077
Baseline SER (D)	−1.39 ± 1.87	−2.44 ± 1.62	−2.24 ± 1.72	0.570
Mean change of axial length (mm)	0.4 ± 0.2	0.5 ± 0.3	0.4 ± 0.2	0.213
Mean change of SER (D)	−0.47 ± 0.92	−0.76 ± 1.05	−0.62 ± 0.97	0.424

SER = spherical equivalent refraction.
