# Supplementary material for: Chinese Eye Exercises and Myopia Development in School Age Children: A Nested Case-control Study
Source: Sci Rep. 2016 Jun 22;6:28531. doi: 10.1038/srep28531 (PMC4916489; doi:10.1038/srep28531)
Supplement: Supplementary Information [file srep28531-s1.pdf]

Chinese Eye Exercises and Myopia Development in School Age Children: A Nested Case-control Study

Meng-Tian Kang MD[1], Shi-Ming Li MD PhD[1 *], Xiaoxia Peng MD PhD[3], Lei Li MD[1], Anran Ran MD[1], Bo Meng MD PhD [1], Yunyun Sun MD [1], Luo-Ru Liu, MD[3], He Li, MD[3], Michel Millodot OD PhD[4], Ningli Wang MD PhD[1 *]

1. Beijing Tongren Eye Center, Beijing Tongren Hospital, Beijing Ophthalmology Visual Science Key Lab, Beijing Institute of Ophthalmology, Capital Medical University, Beijing, China
2. Department of Epidemiology and Biostatistics, School of Public Health and Family Medicine, Capital Medical University, Beijing, China.
3. Anyang Eye Hospital, Henan Province, China.
4. School of Optometry and Vision Sciences, Cardiff University, Cardiff, United Kingdom

Supplement file 1. Standard Chinese Eye Exercises assessment form

Part I Press and knead BL2 (cuanzhu) (8 points)

Item		Score	
Manipulation	Manipulate with thumb	Yes（1 point）	No（0 point）
	Manipulate with fingertip	Yes（1 point）	No（0 point）
	Manipulate in pressing and kneading	Yes（1 point）	No（0 point）
	Manipulation diameter≦2 fingertip	Yes（1 point）	No（0 point）
Acupoint Location	At the median end of the eyebrow	Yes（1 point）	No（0 point）
	In the supraorbital notch	Yes（1 point）	No（0 point）
Strength Effectiveness	Sense of sore (Qi)	Yes（1 point）	No（0 point）
Rhythm	One movement in a beat	Yes（1 point）	No（0 point）

Part II Press and nip BL1 (jingming) (8 points)

Item	Score

Manipulation	Manipulate with index finger	Yes（1 point）	No（0 point）
	Manipulate with fingertip	Yes（1 point）	No（0 point）
	Manipulate in pressing and nipping	Yes（1 point）	No（0 point）
	Manipulation diameter≦2 fingertip	Yes（1 point）	No（0 point）
Acupoint Location	In the depression slightly above the inner canthus	Yes（1 point）	No（0 point）
	Nip bone instead of skin	Yes（1 point）	No（0 point）
Strength Effectiveness	Sense of sore (Qi)	Yes（1 point）	No（0 point）
Rhythm	One movement in a beat	Yes（1 point）	No（0 point）

Part III Press and knead ST2 (sibai) (8 points)

Item		Score	
Manipulation	Manipulate with index finger	Yes（1 point）	No（0 point）
	Manipulate with fingertip	Yes（1 point）	No（0 point）
	Manipulation diameter≦2 fingertip	Yes（1 point）	No（0 point）
	Manipulate in pressing and kneading	Yes（1 point）	No（0 point）
Acupoint Location	Directly below the pupil	Yes（1 point）	No（0 point）
	In the depression of the infraorbital foramen	Yes（1 point）	No（0 point）
Strength Effectiveness	Sense of sore (Qi)	Yes（1 point）	No（0 point）
Rhythm	One movement in a beat	Yes（1 point）	No（0 point）

Part IV Press and knead EX-HN5 (taiyang),scrape orbit (16 points)

Item			Score	
Press and knead EX-HN 5	Manipulation	Manipulate with thumb	Yes（1 point）	No（0 point）
		Manipulate with fingertip	Yes（1 point）	No（0 point）
		Manipulate in pressing and kneading	Yes（1 point）	No（0 point）
		Manipulation diameter≦2 fingertip	Yes（1 point）	No（0 point）
	Acupoint Location	Between the lateral end of the eyebrow and the outer canthus	Yes（1 point）	No（0 point）

		In the depression of the infraorbital foramen	Yes（1 point）	No（0 point）
	Strength Effectiveness	Sense of sore (Qi)	Yes（1 point）	No（0 point）
	Rhythm	One movement in a beat	Yes（1 point）	No（0 point）
Scrape orbit	Manipulation	Manipulate with index finger	Yes（1 point）	No（0 point）
		Manipulate with second phalangeal joint	Yes（1 point）	No（0 point）
		Manipulate in scraping	Yes（1 point）	No（0 point）
		Scrape bone	Yes（1 point）	No（0 point）
	Acupoint Location	On the bony structures of orbit	Yes（1 point）	No（0 point）
		Include the whole orbit	Yes（1 point）	No（0 point）
	Strength Effectiveness	Sense of sore (Qi)	Yes（1 point）	No（0 point）
	Rhythm	One movement in 2 beat	Yes（1 point）	No（0 point）
